# Tuning between Nuclear Organization and Functionality in Health and Disease

**DOI:** 10.3390/cells12050706

**Published:** 2023-02-23

**Authors:** Naresh Kumar Manda, Upendarrao Golla, Kishore Sesham, Parth Desai, Shrushti Joshi, Satyam Patel, Sharada Nalla, Susmitha Kondam, Lakhwinder Singh, Deepak Dewansh, Hemalatha Manda, Namita Rokana

**Affiliations:** 1 Department of Biochemistry, School of Life Sciences, University of Hyderabad, Hyderabad 500046, India; 2 Department of Pediatrics, Division of Hematology and Oncology, Pennsylvania State University College of Medicine, Hershey, PA 17033, USA; 3 Department of Medicine, Division of Hematology and Oncology, Pennsylvania State University College of Medicine, Hershey, PA 17033, USA; 4 Department of Anatomy, All India Institute of Medical Sciences (AIIMS), Mangalagiri 522503, India; 5 Department of Nanoscience, Joint School of Nanoscience and Nanoengineering, University of North Carolina, Greensboro, NC 27401, USA; 6 School of Science, Auckland University of Technology, Auckland 1010, New Zealand; 7 Department of Biological Sciences, School of Science Engineering and Technology, Penn State Harrisburg, Middletown, PA 17057, USA; 8 Faculty of Pharmacy, University College of Pharmaceutical Sciences, Palamuru University, Mahabubnagar 509001, India; 9 Department of Dairy Microbiology, College of Dairy Science and Technology, Guru Angad Dev Veterinary and Animal Sciences University, Ludhiana 141004, India; 10 Department of Tourism Management, Vikrama Simhapuri University, Nellore 524324, India

**Keywords:** nuclear shape regulation, nuclear size regulation, nuclear envelope proteins, nucleophagy, nuclear lamins, nucleopathy, cancer, neurodegenerative disorders, signaling pathways, targeted therapy

## Abstract

The organization of eukaryotic genome in the nucleus, a double-membraned organelle separated from the cytoplasm, is highly complex and dynamic. The functional architecture of the nucleus is confined by the layers of internal and cytoplasmic elements, including chromatin organization, nuclear envelope associated proteome and transport, nuclear–cytoskeletal contacts, and the mechano-regulatory signaling cascades. The size and morphology of the nucleus could impose a significant impact on nuclear mechanics, chromatin organization, gene expression, cell functionality and disease development. The maintenance of nuclear organization during genetic or physical perturbation is crucial for the viability and lifespan of the cell. Abnormal nuclear envelope morphologies, such as invagination and blebbing, have functional implications in several human disorders, including cancer, accelerated aging, thyroid disorders, and different types of neuro-muscular diseases. Despite the evident interplay between nuclear structure and nuclear function, our knowledge about the underlying molecular mechanisms for regulation of nuclear morphology and cell functionality during health and illness is rather poor. This review highlights the essential nuclear, cellular, and extracellular components that govern the organization of nuclei and functional consequences associated with nuclear morphometric aberrations. Finally, we discuss the recent developments with diagnostic and therapeutic implications targeting nuclear morphology in health and disease.

## 1. Introduction

The foundation of life is dependent on the functional stratification of specialized subcellular compartments. In a eukaryotic system, the nucleus forms a distinctive micro-terrain to conceal the genetic material from damaging cytoplasmic enzymes and metabolism and to provide a unique regulatory molecular framework for the genome. The spatial encapsulation of the nucleus by the lipid bilayer forms a physical and physiological intercept between cytoplasmic processes and the genome that regulates them. The construct of the nucleus is collectively furnished by a nuclear envelope along with the underlaying chromatin fiber, intermediate filaments of nucleoskeleton, nucleoplasmic subcompartments and nucleolus. These contractual components collectively impose their own effect on the rigidity, morphology and size of the nucleus [1,2,3]. The nuclear shape and size are also subjected to the layers of cellular regulatory mechanisms, including C/N volume regulators, mechanobiology activated signaling cascades, macro- and micronucleophagy, etc. [4,5,6,7].

Although the nuclear size and morphology varies widely among unicellular and multicellular eukaryotes, its extent is precisely maintained in the individual cell type [8]. However, the nucleus of same cell type may also differ among various growth phases and under different extracellular matrices. It is now understood that the nuclear, cellular or extracellular stimulants which mediate morphological alteration in the nucleus could also modulate gene expression, and therefore, the physiology of the cell [9,10]. The connection between nuclear structure and function has been outlined by many researchers who have categorized the nuclear pathophysiology into some broad groups, such as envelopathy, (a group of disease caused by mutation in genes encoding nuclear envelope proteins), laminopathy (diseases caused by mutations in LMNA gene) and tauopathy (a heterogeneous group of neurodegenerative diseases characterized by deposition of abnormal tau protein in the brain cells), and conferring the major responsibility for malfunctioning nuclear or cellular components to them. The structural aberrations are mostly compelled by abnormalities of nuclear envelope proteins and disorganized nucleoplasmic subcompartments, as well as hindered nucleo-cytoskeletal interactions, nuclear transport and repair mechanism. It is well-known that morphological deformations may alter cell cycle progression [11], chromatin accessibility [12], and the gene expression profile of a cell [13]. Consequently, the genetic rearrangement associated with nuclear aberration could be involved with different types of malignancy, progeria syndromes, neurodegenerative diseases, neuromuscular dystrophy and many other terminal illnesses, as discussed in the following sections.

Nuclear aberrations may be either the cause of a disease or the consequences of cellular events related to the disease. In both of these situations, identifying the factors involved in the modifications could be used to pinpoint the onset of pathogenesis at an earlier stage. Moreover, understanding the connection between the nuclear morphology and the altered cellular and extracellular components could pave the way for designing targeted and effective treatment strategies for many related life-threatening diseases [14].

In this review, we examined the diverse cellular activities associated with regulating nuclear size and morphology. We investigated how the altering or malfunctioning of certain factors affects the shape, size and organization of the nucleus. We have also underlined the concepts involved in specific theranostic approaches for early and targeted diagnosis and treatment of nuclear deformation that accompanies pathogenesis.

## 2. Contribution of Nuclear Constituents in Regulation of Nuclear Morphology

The structural components of a nucleus, such as chromosomes, nucleoplasmic compartments, nuclear envelope proteins and lipid bilayer, are the core elements involved in the regulation of nuclear morphology. Each component has a distinct functionality and approach by which they help to maintain the characteristic shape and size of the nucleus. Here, we will evaluate the mechanisms of individual nuclear constituents that collectively gather to fabricate the controlled morphology of the nucleus in normal and diseased condition.

### 2.1. Nuclear Envelope Proteins

**Figure 1:**
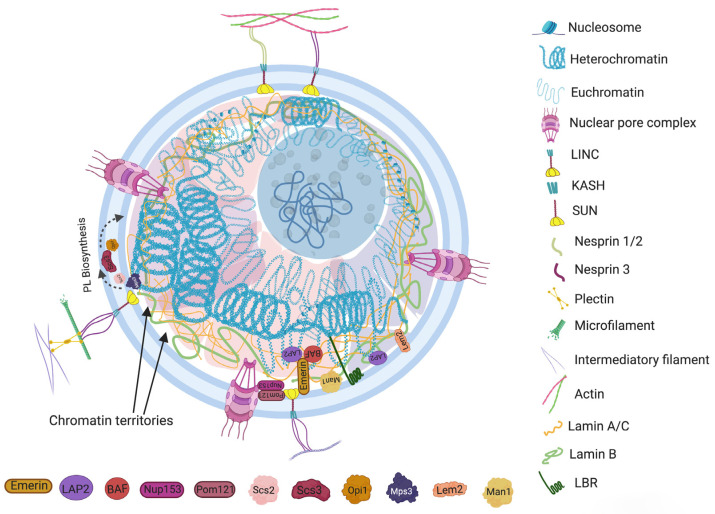
The constituents that contribute to regulation of morphology and characteristic organization of a common eukaryotic nucleus. The major components include the lamin network, nuclear envelope (NE), chromatin and membrane-less nuclear subcompartments. The lamin A/C and lamin B assemble around the inner nuclear membrane. Lamina and colocalized INM anchoring proteins, also known as tethering proteins (i.e., LBR, LEM, BAF, LAP, emerin, etc.), anchor at specific “lamina associated domains (LAD)” of the genome. The nuclear envelope associated components include nuclear pore complex (NPC) proteins (Nup) and the linker of nucleoskeleton and cytoskeleton (LINC) complex. Nup153 along with other membrane curvature sensing proteins (i.e., Pom) equilibrate the membrane shaping forces into the NPC assembly. Nup 153 also co-localize with Sun1 and POM121 proteins, which link the NPCs in nucleo-cytoskeletal network coupling and the mechanobiology of nuclear envelope. LINC physically connects the cytoskeletal framework to the nucleoplasmic filaments. The dynamic intermediate bridge of LINC includes INM anchored SUN domain protein and the ONM embedded KASH motif that interacts with the actin filaments, microtubule and intermediate filaments network using containing proteins, i.e., nesprin-1/2/3 and plectin, etc.; SUN domain proteins, meanwhile, bind to the NPC, lamina and chromatin using several intermediate tethering proteins. Specific INM proteins, such as Mps3, Scs2 and Opi1, contribute to lipid membrane biogenesis during morphological alteration of the nucleus. The nuclear subcompartments (Chromatin territories) are microenvironment created by the concentrate of specific proteins that contributes to the organization of different domains of chromatin fiber into the nuclear volume.

Nuclear envelopes are the structural and physiological interface between the central genomic material and cytoplasm of the cell. The double lipid membrane of the nuclear envelope originates from ER and remains in continuous contact with its network afterward. In contrast to the origin, both the outer nuclear membrane (ONM) and inner nuclear membrane (INM) of the nuclear envelope are enriched with a very distinguished set of proteomes (Figure 1) [15]. These subsets of proteins play key roles in bidirectional nucleoplasmic transportation, maintenance of nuclear architecture, cell cycle control, chromatin organization, gene regulation and DNA repair. The most complex macromolecular assemblies of the nuclear envelope are nuclear pore complexes (NPCs) [16,17,18,19]. NPCs encompass multiple subsets of more than 30 types of nuclear pore proteins called nucleoporins (Nups) [20,21]. The de novo assembly of Nups during interphase starts with the accumulation of Nups in both the outer and inner nuclear membranes, and the subsequent fusion these proteins forms the doughnut-shaped core (consisting of eight spokes arranged around a central channel) of NPC [22,23,24]. The fusion creates an energetically unfavorable and highly curved membrane that surrounds the NPC [25]. Some nuclear-basket-associated peripheral Nups reportedly conserve this membrane curvature by holding the membrane with their amphipathic α-helix domains [26]. Specifically, the synergistic participation of Nup1, Nup60 (yeast) and Nup153 (higher eukaryotes), along with other membrane curvature sensing proteins (Y complex, Nup145, Nup133, Pom34), equilibrate the membrane-shaping forces into the NPC assembly [26,27,28,29]. The colocalization of Sun1 protein with Nup153 and POM121, as well as lamin with the nucleoplasmic basket of NPC, have been discovered in different types of cellular models [30,31,32]. These establishments evidently link the roles of the NPCs in the nucleo-cytoskeletal coupling and mechanobiology of the nuclear envelope; at the same time, the assembly of NPCs could also regulate the nuclear morphology indirectly [33,34,35]. It has been observed that defects in postmitotic assembly of NPCs results in a smaller nuclear size in mammalian cells. The shortcoming of functional NPCs is subsequently reflected in the lower density of NPC at the nuclear envelope, which decreases the nuclear import and localization of lamin proteins, thereby reducing the nuclear size [36]. Furthermore, a study by Kittisopikul et al. on lamina knockout and NPCs knockdown in mouse embryo fibroblast cells confirms the interdependent effect of NPCs and lamins on their respective organization at the nuclear periphery. Knocking down the NPCs situated at close proximity to the lamina (i.e., ELYS, TPR) resulted in a spatial distribution of lamin isoforms and vice versa [37]. The codependent relation between NPCs and lamina suggests that the loss of NPCs’ integrity not only compromises the diffusion barrier but also the morphology of the nucleus, which is linked to the pathophysiology of a number of diseases.

Another macromolecular assembly of NE that spans both INM and ONM is known as the linker of nucleoskeleton and cytoskeleton (LINC) complex. It physically connects the cytoskeletal framework to the nucleoplasmic filaments by forming a dynamic intermediate bridge between them. The elemental structure of LINC complex involves two transmembrane domains, ONM embedded KASH (Klarsicht, ANC-1 and SYNE homology protein) motif and INM anchored SUN (Sad1 and UNC-84 protein) domain protein. KASH motif interacts bidirectionally with SUN domain as well as with the actin filaments, microtubule and intermediate filaments network using different intermediate proteins, i.e., nesprin-1, nesprin-2, nesprin-3, dynein, kinesin and plectin, etc. In different species, at the nucleoplasmic front, various isoforms of SUN domain proteins (SUN1/2/3/4/5, Msp3, kalroid, etc.) also bind to the NPC, lamina and chromatin using several intermediate proteins [38]. The KASH motif is a connecting link between the SUN domainand cytoskeleton. The conserved SUN domain proteins interact with lumen to carry the force aroused between cytoskeletal and nucleoskeletal network [39]. Most importantly, the both components of LINC complex (i.e. KASH motif and SUN domain proteins) physically couple with the plasma membrane and nuclear envelope to provide a mechano-transduction signaling interface between the extracellular/cellular microenvironment and the genome [40]. We will see the molecular route of mechanobiology-mediated nuclear alterations in Section 4. Moreover, the membrane-spanning SUN and KASH motif also interacts with various nuclear envelope transmembrane proteins (NETs) and plays a vital role in maintaining the nuclear architecture [41,42]. Since the LINC complex provides a functional connection between cytoplasmic and nucleoplasmic compartments, any constitutional or compositional change in LINC-associated harnessing proteins could affect chromatin dynamics in the nucleoplasm [43] and cause morphological aberrations in the nuclear envelope [44,45]. The influence of LINC complex on nuclear stiffness could be apprehended by the example of granulocytes. The modified expression level of emerin and its allied network proteins, i.e., lamin A/C, B1 and lamina associated polypeptides 2 β (LAP2β), have been recorded in the nucleus of granulocytes [46]. In addition, the inner membrane anchoring protein Lamin B receptor protein/LBR both mediates the nuclear envelope distortion with underlying heterochromatin and influences the lobular shape of granulocyte nucleus [47]. The resulting cellular malleability provides additional advantage to the cells during migration through narrow intracellular channels [48]. Similar types of adaptations have also been observed in different metastatic cancer cells [49,50,51].

In higher eukaryotes, lamin A/C, lamin B and other associated proteins assemble around the inner nuclear membrane and play a remarkable role in the regulation of nuclear forms and functions. The subtype of lamin B (lamin B1) in particular forms an outer loose meshwork surrounding the tighter, nucleoplasm facing, lamin A/C meshwork, and both isoforms assemble into a distinct but interlinked filamentous network. Cells devoid of lamin isoforms develop an irregular nuclear shape and become susceptible to large scale DNA damage due to a ruptured nuclear membrane [52]. The rigidity of the nucleus is very reliant on lamina and co-localized INM anchoring proteins, also known as tethering proteins (i.e., LBR, Lamina associated polypeptide 2-Emarin-Man1 protein/LEM, Methyl CpG binding protein 2/MECP2, Proline rich protein 14/PRR14, Kugelkern, Kurzkern, etc.) [53]. The divergent expression of these tethering proteins in different cell types or during the cell division and development indicate their distinctive roles in shaping the nucleus [54,55,56,57]. The meshwork of A/C and B type lamins helps in the organization of the chromatin territories by binding to those co-localized tethering proteins that anchor at specific “lamina associated domains (LAD)” of the genome [58]. The study on viscoelastic properties of lamin-null mouse embryonic fibroblast cells revealed that both lamin A and B contribute to nuclear stiffness [59]. Briefly, a manometer-based micropipette aspiration system measured the nuclear resistance or mechanical stability to applied forces on different knockout models of mouse embryonic cells. The cell types with decondensed chromatin increased the viscosity of nuclei. Meanwhile, co-expression of lamin A and lamin B1 increases both elasticity and stiffness and stabilizes chromatin condensation. The lamin A/C predominantly bind to the peripheral heterochromatin via the complex formed with proteins LED, PRR14, etc. [53,60]. The second LBR dependent mechanism is also used to localize the heterochromatin to the peripheral nuclear interior during the cell development and differentiation [60]. The tether proteins contain a long neucleoplasmic, chromatin binding domain with an INM span and a short luminal domain between INM and ONM [61]. The tether between lamina and heterochromatin also provides a docking site for chromatin interacting proteins, including histone and histone modifiers (mostly histone methyltransferases and histone deacetylases) [58]. The INM proteins that have LEM domains bind with lamin and histone deacetylase 3 (HDAC3). The emarin domain anchor to chromatin through barrier to autointegration factor (BAF), a sequence independent DNA binding protein. The LAP2β domain binds to HDAC3 and cKrox (zinc finger transcription factor- Zbtb7b), a DNA binding protein that contain Lamina associating sequence (LAS element). On the other hand, LBR binds to H3/H4 and heterochromatin protein 1 (HP1) [60,62]. The MECP2 and PRR14 protein also connect HP1 with LBR and lamin A/C respectively (Figure 1) [53].

It is well-established that lamins and associated proteins not only form a structural element of the nucleus that maintains the nucleus’s stiffness and morphology, but they also play a crucial role in functional components by regulating gene’s radial position and expression [63,64]. The role of lamins has also been recognized in genome organization and stability, regulation the cell division, DNA replication, DNA repair and the transcription process [65,66]. The absence of these lamin and associated tethering proteins cause modification in organization of peripheral heterochromatin during the cell differentiation and development that may reflect via altered architecture of the nucleus. These facts corroborate the correlation between morphological aberrations of the nucleus and the altered pathophysiology of the cell.

### 2.2. Nuclear Membrane Composition

Nuclear envelopes are one of the most functional organelles of the cell and have many simultaneous operations, including signaling, transport, genome compartmentalization, gene regulation, lipid metabolism, DNA repair and cell division, etc. These functional assortments entirely rely on the composition and physicochemical properties of the lipid membrane. The regulation of fatty acid composition of phospholipids (PL) provides specific biophysical properties, such as fluidity, rigidity or curvature to the membrane, which are required for the maintenance of the integrity and morphology of the nucleus. Interestingly, INM itself could regulate lipid composition with the help of some membrane associated proteins. It was previously noted that INM might host the lipid metabolism to expand the membrane through localized stimulation of phospholipid biosynthesis [67]. Later, numerous proteins involved in the regulation of phospholipid biosynthesis, lipid storage and homeostasis were identified at NE [15]. The lipid homeostasis is a complex and multifactorial mechanism that oscillates between formation of phospholipids and storage lipid using a common precursor phosphatidic acid (PA). Based on the cellular demand, PA could be converted first to diacylglycerol (DAG) and then to the storage lipid triacylglycerol (TAG); in other situations, PA could be converted into cytidine diphosphate-DAG (CDP-DAG) to form structural phospholipids.

Furthermore, in depth investigation outlines the contribution of specific INM associated proteins in lipid membrane biogenesis during morphological alteration of the nucleus. In response to the growth signals during stationary phase, a conserved PA-phosphatase Pah1 generates DAG from PA at nuclear membrane subdomain connected with storage lipid droplet. During NE growth, the activity of Pah1 is regulated by Nem1-Spo7 complex, which redirects PA towards phospholipid synthesis and membrane expansion [68]. Many advanced studies in this line also suggest that INM localized lipid modifying proteins could also modulate nuclear morphology by transcriptional regulation of lipid synthesis genes. An interesting study by Friederichs and co-workers revealed that the nuclear morphology in budding yeast can be altered by a monopolar spindle 3 (Mps3), which is lipid remodeling mechanism that uses the activity of SUN protein [69]. The previous knowledge describes Mps3 protein as an initiator of spindle pole body (SPB) duplication and a mediator for tethering SPB to the membrane. The depletion of this protein also causes overproliferation of the inner nuclear membrane due to accumulation of abnormal amounts of polar and neutral lipids; it also inhibits the biosynthesis of sterols into the membrane [69]. It was proposed that Mps3 promotes membrane rigidity by influencing the balance between diacyl glycerol (DAG) and phosphatidic acid (PA). Further exploration of the underlying mechanism by Ponce et al. explained that Mps3 is uniquely positioned at INM to perform along multiple pathways. Its N-terminal remains in the nucleoplasm to anchor the telomeres close to the nuclear periphery, whereas the C-terminal situated in the lumen could mediate lipid metabolism. The authors reasoned that a link between Msp3 and Scs2 (a phospholipid biosynthesis and lipid trafficking protein) could be a possible mechanism for this behavior [70]. Scs3 is localized at ONM and has the affinity to bind with a transcriptional corepressor of the phospholipid biosynthesis enzyme gene Opi1 [71]. Using the connection with Scs2, Msp3 could mediate transcriptional control of lipid synthesis at the nuclear periphery (Figure 1). Similarly, Romanauska and Kohler also postulated the role of storage lipid droplet associated INM protein in the Opi1 mediated transcriptional circuit regulation [72]. However, further validation of theory is needed before drawing concrete conclusions.

The nuclear aberration during growth, division or stress that leads to membrane deformation could also be regulated by remodeling the membrane properties and recruiting specific lipid species in the nuclear envelope. For example, Hwang et al. noticed that the morphological abnormalities in the aneuploid yeast and human cell nucleus could be suppressed by accumulation of long-chain base fatty acids in the membrane [73]. The extra chromosome number in aneuploid yeast generates biophysical stress on the nuclear membrane. To release this stress, these single chain amphipathic molecules provide tight packaging and high curvature to the membrane [73]. Evidently, maintaining dynamic nuclear envelope during different physiological and environmental conditions requires recurrent remodeling of the membrane lipid profile. It is not yet understood how the nuclear membrane sensitizes these biophysical stresses and saves the nuclear integrity via alteration of phospholipid metabolism.

### 2.3. Genome Organization

The organization of the genome within the nucleus is a nonrandom process. The second level arrangement of the genome contains euchromatin and constitutive or facultative heterochromatin that gives rise to some advanced assemblies, such as chromosome loops, topological associated domains (TADs, fundamental units of three-dimensional (3D) nuclear organization), lamin associated domains (LADs, heterochromatin located adjacent to lamina), nucleolar associated domains (NADs, heterochromatin located adjacent to the nucleolus) and chromosome territories. It is also known that the nuclear arrangement of chromatin is somehow related to the morphology of the nucleus [58,74]. The role of chromatin in sizing and shaping the nucleus is very intricate and diverse. However, it is widely understood that chromatin contributes to nuclear morphological regulation by (i) interacting with nuclear envelope via the LAD/NAD binding domains of INM integrated proteins and (ii) altering the biophysical properties of heterochromatin.

In addition to nuclear envelope assembly, the biophysical state of constitutive and facultative heterochromatin largely influences the rigidity, shape and size of the nucleus [2,75]. Numerous studies have explored the role of ‘chromatin packing’ in nuclear morphology. A direct investigation was completed by Stephens et al. on chromatin decompaction of mammalian cells using histone deacetylase and histone methyltransferase inhibitors [2]. The study showed that an increase in the ratio of euchromatin caused softer nuclei and nuclear blebbing, which was independent of the involvement of lamins. The deformation was reversed after treating the cells with histone demethylase inhibitors. It was suggested that decompacted euchromatin might be mechanically weaker than heterochromatin, or that the altered chromatin state could cause a loss of chromatin lamina connection and nuclear rigidity [2]. In search of mechanisms involved in the mediation of nuclear volume through chromatin compaction, Furusawa et al. investigated the interaction of heterochromatin and a nucleosome binding protein HMGN5 [76]. HMGN5 is found at the periphery of the nucleus and is bound to the underlying nucleosome without any sequence specificity. The overexpression of *HMGN5* in transgenic mice decreased chromatin compaction by reducing the interaction between histone H1 and chromatin. Decompaction of chromatin leads to a decrease in nuclear rigidity and a subsequent increase in nuclear blebbing [76]. Hence, the structure of the nuclear envelope and the disseminated genetic material inside it are not at all independent from each other. Therefore, the constant mobile states of the genome have a significant impact on nuclear mechanics.

For instance, Imbalzano et al. have reported the effect of the ATPase dependent chromatin remodeling enzyme BRG1 on nuclear structure. Inhibition of BRG1 activity resulted in irregular nuclear morphology [75]. Corresponding to this, Wang et al. found that increased activity of WDR5 (WD repeat domain 5), an epigenetic modulator of H3K4 methylation, resulted in less compacted euchromatin in acute lymphoblastic leukemia (ALL) cells [77]. The observations indicate that chromatin associated alternation of nuclear morphology in certain conditions could be induced by altered biophysical stress into the nucleoplasm.

### 2.4. Nuclear Subcompartments and Nucleolus

Nucleoplasms are among the very eventful and crowded niche of the cell that provides a common working platform to several types of heterogeneous components. It includes chromatin attached proteins and other nuclear bodies, such as nucleoli, Cajal bodies, promyelocytic leukemia (PML) bodies, speckles, paraspeckles, polycomb bodies and histone locus bodies. The consortia of nuclear bodies combine to make the nuclear matrix, which is responsible for organizing different domains of chromatin fiber into the nuclear volume [78]. The microenvironment created by the concentration of specific proteins is referred to as membrane-less nuclear subcompartments (Figure 1). Some components of nuclear subcompartments could also contribute to the structural organization of the nucleus. For example, Morelli and coworkers have observed that the aberrant expression of heat shock protein B2 (HSPB2), which is a nuclear subcompartment protein, in myoblast cells could cause impaired LMNA-SUN2 anchoring at the nuclear envelope, thereby disrupting NE integrity [79]. The findings also stimulated reasonable thoughts about the impact of the nucleolus on nuclear morphology. Nevertheless, a multiprotein mixed lineage leukemia 4 (MLL4)–complex of proteins that is involved in epigenetic modification was also found to play a crucial role in preserving the mechanical properties of the nucleus by maintaining the equilibrium between chromatin and associated biomolecular condensates [80]. In the congenital disorder Kabuki syndrome, a haploinsufficiency causes a loss of function of MLL4 that affects chromatin liquid–liquid phase separation and alters the assembly of transcriptional condensates and transcriptional regulation of cohesion and condensing genes. The mesenchymal stem cell-based Kabuki syndrome model showed that the impaired chromatin compartmentalization due to loss of function of MLL4 could increase mechanical stress through increasing the chromatin compaction and nuclear stiffness, followed by altering the nuclear architecture in the disease condition [80].

The nucleolus is the most prominent nuclear subcompartment and covers almost one third of the nucleoplasm’s peripheral space. Its size varies during growth, and both normal and cancer cells proliferate due to the increased demand for ribosome biogenesis [81,82,83]. Almost any type of cancer exhibits abnormalities in the number and shape of nucleoli due to overactivated ribosome biosynthetic core machinery. However, it would be interesting to know whether nucleolus could have any influence on nuclear morphology in any of these conditions. The little research conducted on this topic have shown that a nucleolus has the ability to sequester the nuclear envelope to avoid nuclear morphological disruption [84].

The direct interaction between the nuclear envelope and nucleolus was explored by some researchers. A study on breast cancer cells revealed that depletion of the nuclear envelope protein *SUN1* induced nucleolus enlargement [85]. It is already known that INM anchored and associated proteins contribute to maintaining nuclear envelope integrity and morphology. Sharing nuclear envelope proteins to maintain nucleolar and nuclear morphology was also observed by Sen Gupta and Sengupta [55]. The authors reported the independent role of lamin B2 at the nucleolus and nuclear envelope. Collectively, the N-terminal head domain of lamin B2 interacts with the nucleolar proteins nucleolin and nucleophosmin, whereas the C-terminal tail domain makes contact with the nuclear envelope. Depletion of lamin B2 caused morphological abnormalities in both the nucleolus and the nuclear envelope [55]. These studies indicate the presence of common mechanisms which regulate both nucleolar and nuclear morphology. Indeed, there is not sufficient information to know where there is any substantial correlation between the regulation of the nucleolus’s morphology and the nucleus. If yes, then how and in which direction are these mechanisms induced (from nucleolus to nucleus or from nucleus to nucleolus) and what are the exact regulating factors between them? These queries need to be addressed to resolve the ambiguity and to present a clear picture.

### 2.5. Nucleus and Cytoplasmic Components

The nucleus is a largest organelle and the center of essential genetic and regulatory activities of the eukaryotic cell. Constant physiological communication among the nucleus and other cellular components, such as mitochondria, ER, vacuoles, peroxisomes, plasma membrane, lipid droplets and cytosol, maintains the cellular homeostasis [86,87]. Strikingly, the direct physical interconnection involving specific tethering contacts has also been recognized among the membrane-bound organelles [88]. In this context, the involvement of reticulon (Rtn), an ER membrane stabilizing protein, is reviewed by Mukherjee et al. [1]. The increased activity of Rtn is observed with a decreased nuclear size in many cell types [89,90,91]. Beside macro-organelles, the cytoskeleton makes up a significant portion of the cytoplasm and plays an important role in nuclear positioning and regulation of its morphology.

**Figure 2:**
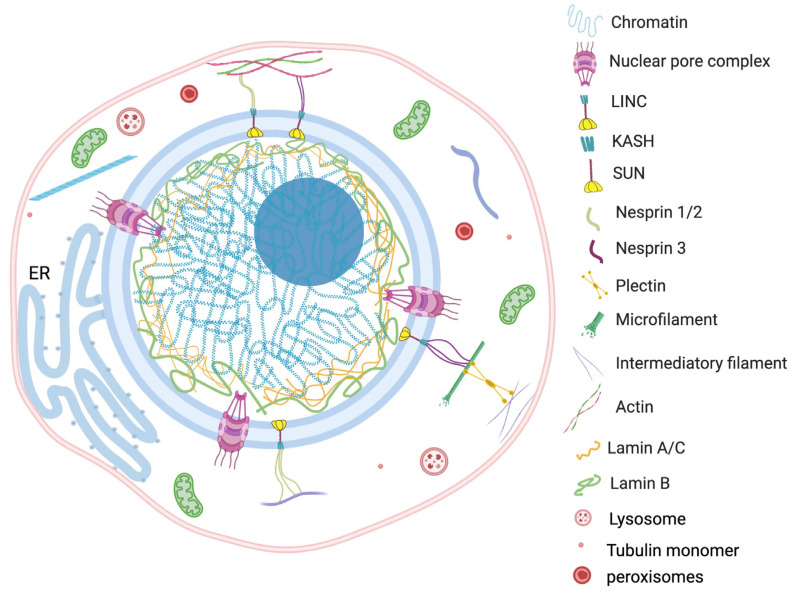
Nucleus contexture and interaction between nucleus and cytoplasmic content. The mechanical transduction of external forces affects nuclear morphology through interaction between nuclear matrix and cytoskeleton. The figure represents specific bonding between cytoplasmic macromolecules (actin, tubulin pectin, etc.) and nuclear LINC complex, lamins, SUN protein, KASH motif and nesprin protein. The physical interconnection involving the specific tethering contact of the nucleus with the membrane-bound organelles, such as ER, also plays an important role in nuclear positioning and regulation of its morphology.

Since the nucleus is the largest and most vigorous organelle of the cell, the organization or reorganization of the cytoskeleton quickly transmits the cellular stress to the nucleus. For example, findings of Monroy-Ramírez and coworkers established that aberrant binding of tau protein and tubulin alters the radial organization of cytoskeleton to the thick ring type arrangement at peripheral and perinuclear sites [92]. The rehabilitated nuclear–cytoskeleton assembly causes enlargement and lobulation of the nucleus followed by functional abnormalities into the cell. The externally applied tension transfers to the nucleus via the actin filament anchoring LINC complex. The direct connection between actin cytoskeleton and nuclear morphology was observed in human melanoma cells by Colón-Bolea et al. [93]. The nuclear shape alteration in invasive melanoma cells was orchestrated by alteration in the connection between the tubulin cytoskeleton and LINC complex using a RHO GTPase (RAC1)-mediated mechanism [93]. The concept is further corroborated by Lu et al., who demonstrated the consequence of disruption in connection between a KASH motif containing proteins and an actin network [94]. A multivariate KASH motif containing protein, Nesprin, interacts with the actin cytoskeleton covering the outer nuclear membrane. The study revealed that Nesprin 1/ Nesprin 2 consists of a specific N-terminal actin binding domain (ABD) which is involved in actin mediated nuclear shape regulation. The overexpression of Nesprin 2 ABD leads to increase in nuclear area, but replacing it with a mini-isoform of Nesprin 2 that lacks the long rod segment produces smaller nuclei [94]. The authors proposed that an interchain association of Nesprin produces a basket-like protein network which has a key role in effective transduction of nuclear and cytoplasmic forces. The nuclear shape is the net outcome of external (cytoskeleton) and internal (microfilaments, lamina, genome) generated forces from opposite sides of the nuclear envelope (Figure 2).

Furthermore, research into isolated nuclei has also revealed that nuclei are able to resist force by adjusting their stiffness in the direction of the applied tension [95]. This acclimatization is completed by phosphorylation of tyrosine residues on the emerin protein followed by rearrangement in the LINC-lamin A/C connections. In addition to reinforcing its rigidity, nuclear membrane tension is sometimes lowered to dissipate the mechanical energy. Recently, Nava and coworkers found that Ca^2+^ influxes from ER to nucleoplasm are enhanced to induce nuclear softening during mechanical stretch conditions [74]. This is thought to be a defense mechanism designed to prevent the mechanical damage of genetic material by changes in the Ca^2+^ dependent chromatin rheology. Release of Ca^2+^ reduces the association between lamina and H3K9me3-marked heterochromatin, and subsequent nuclear softening is required to insulate the genetic material [74]. Hence, this untethering of chromatin from the INM under cytoskeletal forces could result in a highly deformable nucleus [96]. Further exploration of nuclear structural and physiological harmony under the influence of physical forces reveals that uneven deformation of the nucleus enhances the expression of some mechanosensitive genes [11,97,98,99]. These studies found that deformation of nucleus due to force transmission causes localization and activation of some mechanosensitive transcription activators (i.e., YAP, AP1, TEAD) in the nucleus. This connects the role of nuclear morphological aberrations in cell fate switch and the development of pathogenicity.

## 3. Functional Consequences of Nuclear Aberrations

**Figure 3:**
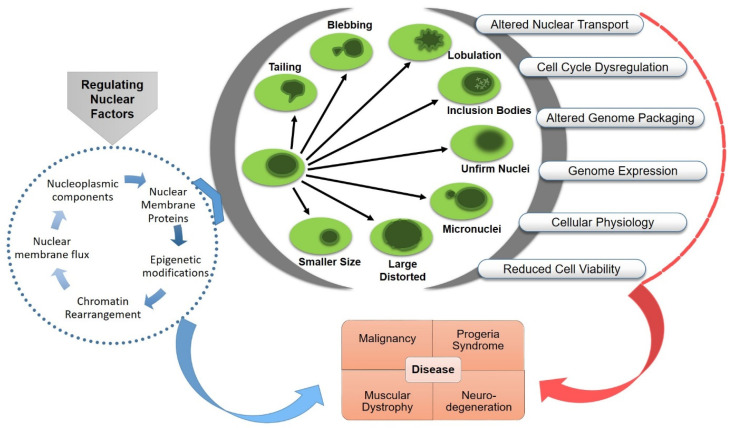
Nuclear aberrations and functional abnormalities. Pathophysiology of related diseases and possible targets for the treatment.

Any morphological aberration of the nucleus could be rooted in functional abnormalities, including instability of genetic material, aneuploidy, micronuclei formation, altered gene expression and metabolic dysregulation. Nuclear pathophysiology is categorized into broad groups based on the major responsible malfunctioning component, such as envelopathy (nuclear envelope proteins that are involved in fundamental nuclear functions, such as gene transcription and DNA replication, cause human diseases through inherited or de novo mutated proteins cause human diseases, called “nuclear envelopathies”), laminopathy (diseases caused by mutations in LMNA gene, called “laminopathies”) and tauopathy (a heterogeneous group of neurodegenerative diseases characterized by abnormal metabolism of misfolded tau proteins (tau prions) which eventually results in massive loss of brain cells). Such structural aberrations affect the operational activities of the nucleus and causes devastating impact on human health, including oncogenesis, aging disorders, neuronal or muscular dystrophy or cardiomyopathy [100,101,102,103]. The pathophysiological significance of nuclear deformation has been studied exponentially in human and animal subjects, which is reflected by a tremendous number of publications in this field. Here, we will examine the cellular cause or consequences of nuclear deformation relating to physiological disorders (Figure 3).

The relation between nuclear deformation and progression of physiological defects are widely studied in cancer cells. In contrast to normal cells, the tumorigenic cell’s nucleus shows an unusual size and a floppy and irregular appearance due to fragmented, lobulated or deep invading outline [104,105]. The altered structural mechanics provide plasticity and increased invasion properties to metastatic cells; they also induce chromatin remodeling and cell cycle regulation in primary oncogenic cells [100,106]. Mutations in a large range of NE proteins are frequently observed in different types of cancer cells. It has been noted that the deregulation of lamin or emerin proteins could predispose mechanical distress that compromises nuclear compartmentalization and nuclear envelope integrity in cancer cells [107,108] and causes DNA damage in skeletal muscle cells [109]. Most types of the cancers show aneuploidy during the progression of carcinomas. The chromosomal instability of cancer cells also found associated deformation of nuclear envelope. The mechanistic study on ovarian cancer cells has revealed a mechanism mediated by suppression of the GATA6 transcription factor followed by loss of the nuclear envelope protein emerin [110]. Furthermore, Nader et al. explored how nuclear deformation in cancer cells leads to chronic and sublethal damage of genomic DNA. The study recorded the presence of an ER membrane-associated exonuclease, TREX1, in the deformed nucleus of tumor cells. The TREX1-mediated DNA damage again promoted tumor growth and invasion by leading aberrant invasiveness in the tumor cells [111]; the nuclear instability caused by altered expression of NE proteins is required for tumor aggressiveness in different types of cancer [50,112]. The laminopathy-linked nuclear envelope fragility sometimes leads to abnormal nuclear division and formation of unstable micronuclei that have small genome fractions can cause aneuploidy, a common feature in oncogenic cells [113].

The rearrangement of transenvelope components, such as LINC complexes and NPCs, are required for the coordinated cell migration and attachment of invasive malignant cells [114]. Furthermore, the altered arrangement of these nuclear envelope proteins could also modulate the genome organization that changes the nuclear mechanophysics and gene expression profile. For example, atypical Nup98 protein contributes to morphological alteration by affecting the lamina and lamina-associated polypeptides 2α (LAP2α) in leukemia cells [115]. The formation of chimeric protein involving NUP98 and transcription factors, such as homeodomain (HD), were observed to induce morphological alterations of the NE in acute myeloid leukemia (AML) cells (Table 1) [115]. The aberrant NE phenotypes include lobulation due to altered chromatin organization, relocalization of A and B lamins and alteration in lamin A associated LAP2α protein. The LAP2α is a networking protein that interacts with nucleosome binding proteins, thereby affecting chromatin distribution and NE organization associated with malignant transformation. The similar protein has also been reported to be involved in epigenetic regulation of gene expression using histone modifying complex in yeast, drosophila and human leukemia cells [116,117]. Similarly, a nuclear importer family protein, karyopherin α7 (KPNA7), is expressed at higher level in cancer cells. The intensity of KPAN7 protein affects the organization of lamina and nuclear morphology. Interestingly, it also has a critical role in the organization of mitotic spindles and acts as an important element in cancer cell proliferation [118]. On the other hand, some of the nuclear proteins have been found to regulate cell growth, apoptosis, and differentiation in cancer cells using components of cell signaling pathways. For instance, Kong and colleagues observed a correlated change in the level of lamin A/C and PI3K/AKT/PTEN pathways in prostate cancer cells [119]. A large-scale study on primary lung cancers uncovered that larger distorted nuclei of tumor cells have significant association with the altered expression of cell cycle checkpoint protein p53 and DNA repair protein p16INK4A [120]. The spontaneous link between signaling proteins and nuclear deformities is recorded in numerous studies (reviewed in [114,121]). However, the precise connecting mechanisms by which cancer cells stimulate mechanotransduction signaling to maintain self-sustained proliferation remain elusive.

Lamin A farnesylation, which is key to almost all cellular defects and nuclear deformations, is also a principal prognosis component of premature aging or progeroid syndromes. Progeroid syndromes are terminal genetic disorders characterized by an accelerated aging process due to a decline in physical and physiological function at early age [122]. Aging nucleus shows evident structural and molecular changes, including nuclear membrane lobulation and detachment, altered nuclear transport, altered genome compartmentalization and packing and an increase in transposable element transcripts and nuclear inclusions [123,124,125]. The nuclear defects in progeria syndrome are caused by mutations in the LAMA gene. For example, in Hutchinson–Gilford Progeria Syndrome (HGPS), mutations in exon 11 of the *LMNA* gene alters its splicing pattern and results in an in-frame deletion at C-terminus in prelamin A that produces a protein which is 50 amino acids shorter. “Progerin,” an altered prelamin A protein, interrupts the function of normal nuclear lamina at the nuclear periphery [126]. The progerin-induced irregularities include nuclear envelope blebbing, relaxation of peripheral heterochromatin, altered epigenetic modifications and, thus, gene expression [124,127]. Even after correct expression of the LMNA gene, the defects in post-transcription modification of prelamin A protein may cause several premature aging diseases, including HGPS, mandibuloacral dysplasia syndrome (MAD) and restrictive dermetopathy (RD). A membrane zinc metalloprotease, ZMPSTE24, is a crucial tool for biogenesis of the lamin A scaffold protein. For the prelamin A substrate, encoded by LMNA must be farnesylated and carboxymethylated at C-terminal CAAX motif [128]. Recessive LMNA and ZMPSTE24 mutations impede the prelamin A post-transcriptional modifications mediated by the ZMPSTE24 metalloprotease and cause cardinal nuclear morphological dysfunctions (Table 1). Moreover, similar nuclear disorders are recorded in multiple cancers, nucleopathies associated with muscular cells (Emery–Dreifuss muscular dystrophy, EDMD), neurons (Alzheimer’s disease and Parkinson’s disease), adipose (familial partial lipodystrophy), and myofibroblasts (Table 1). The dissimilar genesis of different types of nucleopathies provides inclusive information for disease prognosis. For example, mutations in a range of LINC complex components and LMNA alters the nuclear envelope plasticity in EDMD disease [101]. Another observation was recorded in a cardiomyopathy and muscular dystrophy mutant model of mice embryonic fibroblast. The study revealed that amino acid substitution in LMNA caused an increase in nuclear size and dilution of heterochromatin near the lamina without altering the nuclear morphology [129]. The proposed pathogenicity mechanism suggested that mutant lamin A/C variant leads to chromatin organization and gene expression, followed by altered cellular mechanotransduction. In myofibroblast emerinopathy, the altered emerin function causes failure of perinuclear actin fibers assembly [103]; in Alzheimer’s disease, however, tau protein-induced nuclear envelope invagination coupled with lamin B dysfunction causes neuronal death [102]. An age dependent aberrant inclusion of two RNA binding proteins, the Musashi and tau proteins, are also reported to cause nuclear transport, chromatin remodeling and nuclear lamina formation in Alzheimer’s disease [130]. The progression of Parkinson’s disease, the most common age-related movement disorder, is diagnosed by degradation of dopaminergic, nigrostriatal neurons, which is reportedly caused by multiple factors affecting cellular homeostasis. Among them, toxic accumulation of a presynaptic protein α-synuclein and the missense mutation of Leucine-rich repeat kinase 2 (LRRK2) reportedly contribute to PD related motor symptoms by causing dopamine transmission dysfunction among the neurons. The LRRK2 deficiency is also correlated with nuclear hypertrophy, nuclear invagination and dendritic atrophy during aging [131,132]. The nuclear morphological and functional alteration in brain neurons is also a hallmark of Huntington’s disease, another neurodegenerative disorder. The disease mechanism studies have established a relation between altered lamin B levels followed by altered nucleoplasmic transport, perturbation in nuclear lamina heterochromatin organization and altered nuclear morphology HD specific brain neurons [133].

Associations have been found between many important genetic or inherited diseases and an array of nuclear deformations. For instance, in Down syndrome, the extra copy of chromosome 21 affects the nuclear organization following epigenetic rearrangements that increase heterochromatin and reduces global transcription level, hinders the nucleoli fusion pattern that increases the number of nucleoli and influences the pre-mRNA splicing that reduces the number of Cajal Bodies [134] (Table 1). Hence, complete knowledge of molecular mechanisms activated by nuclear deformation in such physiologically challenging conditions will be instrumental for strategic management of the diseases.

**Table 1:** Human diseases pertaining to nuclear aberrations.

S.No.	Disease	Associated Nuclear Abnormalities	Cause	Reference
	Cancers			
1.	Breast cancer	Deformed nuclei; nuclear envelope rupture	*TREX1*-dependent DNA damage	[111]
		Aberrations in nuclear morphology and aneuploidy	Loss of A-type lamin expression	[107]
2.	Lung cancer	Larger nuclei with distorted nuclear outlines	High levels of p53, low levels of p16INK4	[120]
3.	AML	Morphological alterations in the nuclear envelope affecting the nuclear lamina and the LAP2α	Nup98 fusion proteins-associated aberrations	[115]
4.	MDS	Abnormal nuclear morphology	Loss of lamin B1 (*LMNB1*)	[64]
5.	Colon cancer	Altered nuclear shape	Loss of lamin A/C expression	[135]
6.	Ovarian cancer	Nuclear protrusions and formation of micronuclei	Suppression of lamin A/C produced aneuploidy	[136]
		Nuclear deformation and aneuploidy	Loss of GATA6 and emerin	[110]
7.	Cervical Cancer	Distortion of nucleolar and nuclear structures	Depletion of nucleophosmin	[137]
	Progeria Syndromes			
8.	HGPS	Increased nuclear stiffness and sensitivity to mechanical strain	LMNA gene mutations and expression of a mutant protein “progerin” into nucleus	[138]
9.	Mandibuloacral Dysplasia	Independent nucleus-like structures; irregular shaped nuclei with nuclear membrane invaginations; doughnut-shaped nuclei, large protrusions (“buds” or “blebs”)	Mutations in *LMNA*, *ZMPSTE24* genes, etc.	[139,140,141,142]
10.	Atypical-Werner syndrome	Irregular nuclear shape, blebbing and chromatin disorganization	*LMNA* gene mutation	[143,144,145]
11.	WRS/ Neonatal progeroid disorder	Enlargement of nuclei and nucleoli	Accumulation of small RNAs in the nucleoli	[146]
	Neurodegenerative Disorders			
12.	Alzheimer’s disease	Disruption of nucleoskelaton, nuclear envelope lobulation, smooth nuclear exterior, tubular invaginations of the nuclear envelope	Accumulation of lamin-rich meshwork at inner nuclear membrane; soluble nuclear aggregates of RNA binding proteins (Musashi and tau)	[130,147]
13.	Parkinson’s disease	Nuclear fragmentation and condensation. Enlargement of nucleus and nuclear invagination	Deposition of α-synuclein aggregates, multiple missense mutations in Leucine-rich repeat kinase 2 (*LRRK2*) gene	[132,148,149]
14.	Huntington’s disease	Altered nuclear morphology and nucleocytoplasmic transport disruption	Presence of a faulty gene (mhTT) on chromosome number 4, increased lamin B1 levels	[133]
	Neuromuscular Diseases			
15.	EDMD	Lobulation and focal widening of the space between inner and outer leaflet of the nuclear envelope, significant nuclear volume alteration, more spherical nuclear shape, nuclear envelope rupture	Mutations in lamin A, *SYNE1*, nesprin-1 and -2, *SUN1* and *SUN2* and *EMD* (or *STA*) gene	[109,150,151,152]
16.	Dilated cardiomyopathy	Aberrant nuclear morphology and size	Mutations in the *LMNA* gene	[153,154]
17.	Congenital muscular dystrophy	Nuclear envelope rupture; mechanically weak nuclei; irregular/elongated nuclei with multiple herniations	Mutations in the *LMNA* (R249W) gene	[109,155]
	Genetic Disorders			
18.	Down Syndrome	Reduced nuclear size; changes in chromatin configuration; nucleoli and Cajal bodies; alterations in the nuclear architecture	An extra partial copy or full copy of chromosome 21 (trisomy)	[134]
19.	PHA	Neutrophils with dumbbell-shaped, bilobed nuclei; a reduced number of nuclear segments; and coarse clumping of the nuclear chromatin, loss of nuclear lobulation in granulocytes. Hypolobulated nucleus in neutrophils	Mutation in lamin B receptor	[47,156,157]
	Other Rare Disorders			
20.	Kabuki syndrome	Altered chromatin liquid–liquid phase separation, nuclear mechanical properties and architecture	Haploinsufficiency of *MLL4*	[80]
21.	Restrictive dermopathy	Massive intranuclear accumulation of wild-type Prelamin A	Heterozygous mutations in *ZMPSTE24* gene; and de novo mutations of the *LMNA* gene	[158,159]

AML-Acute Myeloid Leukemia; LAP2α-Lamina-associated polypeptide 2α; MDS-Myelodysplastic Syndrome; HGPS-Hutchinson–Gilford Progeria Syndrome; WRS-Wiedemann–Rautenstrauch Syndrome; EDMD-Emery–Dreifuss muscular dystrophy; PHA- Pelger–Huet Anomaly.

## 4. Therapeutic Approaches Targeting Nucleus in Disease and Identification of Potential Diagnostic Biomarkers

The shape of the nucleus impacts the functional status of the cell. Although the majority of cell types have either a spheroid or ovoid nucleus, different cell types can have different nuclear shapes, such as lobed, spindle shape, etc. These varied nuclear shapes have a definitive role in the transcriptional or functional activity of the cell. The human granulocytes are a good example of the need for varied nuclear shape to perform different functions. Mature neutrophils have multilobed segmented nuclei separated by thin filaments of nucleoplasm facilitating the flexibility necessary for them to pass through small gaps in the endothelium and extracellular matrix more easily. The bilobed circulating monocyte nuclei become more rounded following recruitment into tissues that further differentiate into macrophage.

The assembly of the nucleus is dynamically organized to adjust its shape and size to maintain homeostasis during different phases and needs of the cell. It is a common phenomenon of cellular functionality in which alterations in morphology happen in response to a modification in the cell’s physiological or structural environment. These morphological alterations are vital to maintain optimal functioning of the nucleus during growth and the cell’s changing needs under stress. However, the same has also been correlated with the development of cancer and several other neuronal or muscular disorders (Table 1) [160]. Altered mechanical properties of nuclei are associated with altered cell behavior and disease. Here, we sought to determine the nuclear deformation-based pathogenesis and possible utility of such knowledge in the development of therapeutic approaches.

Nuclear morphometry plays a significant role in the histopathological and cytological diagnosis of many diseases. For instance, a 35-month follow-up study on osteosarcoma patients revealed that nuclear morphological parameters, such as area and shape, could be applied to identify which patients had a good prognosis [161]. It was also recorded that patients with large and round tumor nuclei had better outcomes then patients with small and polymorphic nuclei. Nuclear morphological changes include alterations in size, shape, margins (grooves/molding/convolutions/thickening), shifts in chromatin pattern, enlargement of nucleoli and perinucleolar space. Morphometry and image analysis techniques are helpful to characterize the size and shape of nuclear substructures, such as nucleoli, nuclear membranes and chromatin granules. Intranuclear informatics have been developed by combined application of fluorescence microscopy, image processing and statistical analysis using specific computerized nuclear morphometric methods [162].

Irregularities in nuclear size, shape and chromatin texture are often correlated with altered gene organization and expression in tumor cells [11]. The remedy of such complications is completely dependent on early-stage diagnosis, when the disease is less destructive and treatment is more effective. Thereby, specific structural aberrations, including blebbing, development of nucleoplasmic reticulum, altered size and number of nucleoli and changes in nuclear rigidity have been used as important diagnostic standards to determine the type and stage of disease [104,163,164]. For instance, Antmen et al. identified differences in the mechanical properties of breast cancer cells at three different disease states, including benign, malignant noninvasive and malignant highly invasive breast cancer cells [165]. The three cell types showed nuclear deformability in order to progress their malignancies when observed using a scanning electron microscope (SEM) and fluorescence micrograph over a specific micropatterned substrate film. The increased nuclear deformation was also correlated at the molecular level with suppressed expression of Lamin A/C and Nisprin-2 genes in respective cells [165]. There are several quantitative imaging techniques that could identify the irregularities in nuclear shape (area, diameter and perimeter), nuclear contour ratio (circularity or lobulation), boundary curvature and elliptic Fourier coefficient ratio (deformation) with higher accuracy [166]. Along with imaging techniques, the presence of circulatory nuclear matrix proteins (e.g., NMP22, NuMA, lamin B1) in the body fluids (plasma, urine, saliva, etc.) is used as a biomarker for diagnosis and prognosis of many cancer types, including prostate, bladder, colorectal, hepatic, head and neck cancers [167,168,169,170,171]. Recently, Wu et al. reported that the nucleus morphology features measured in more than 30,000 single-cell-derived clones from the parental breast cancer cells exhibited distinct and yet heritable traits associated with genomic and transcriptomic phenotypes [172]. These findings highlight the significance of nuclear morphometric analysis through digital pathology combined with multiomics (i.e., single-cell genomics, transcriptomics) for improved diagnosis and prognosis of individual cancer patients [173]. In vitro analysis of morphological features could offer an effective and affordable method to reveal the intratumoral heterogeneity, thereby improving the overall disease prognosis and survival.

The nuclear–structural abnormalities-based prognostic or diagnostic approach has been further extended for the development of targeted and personalized treatment strategies [174]. Moreover, the histological measurement of nuclear abnormalities may also be used as a marker to access the efficacy of those treatments. The study by Stephens et al. on lamin B1 and A mutant progeria model showed a similar concept [2]. The authors established that increases in heterochromatin level-based nuclear stiffness using histone demethylase inhibitors improved nuclear morphology by decreasing the number of blebbed nuclei in progeria cells [2]. Relatedly, Dou et al. have also suggested that inhibition of LC3-lamin B1 interaction protects cells from tumorigenesis by preventing lamin B1 loss and attenuating oncogene-induced senescence in primary human cells [175]. Targeting the signaling pathways regulating nuclear morphology has also been suggested by some researchers in a few disease models. For example, two centromere binding proteins namely transforming acidic coiled-coil (tACC) domain-containing protein and tuberous sclerosis 2 (tSC2) play an important role in nuclear morphology management [176]. Both proteins are regulated by Akt-mediated pathways which could be used as key therapeutic target in abnormal cellular growth [177]. tSC2 is a tumor suppressor and gatekeeper protein that functions as GTPase activating protein in association with tSC1 protein. Meanwhile, tACC is a centromere binding protein that also has a significant role in maintenance of nuclear membrane structure and cell division after binding with tSC2. The direct correlation between the lamins, NPCs and tumor suppressor protein p53 was elucidated by Panatta et al. very recently [178]. Their observation of p53 depleted mouse pancreatic ductal adenocarcinoma cell revealed that p53 regulates the expression of nuclear component genes, including *Lmnb1*, *Tmpo*, *Nup205*, *Nup107*, *Nup85* and *Nup35*. The p53 protein indirectly represses these target genes using a cell cycle regulating protein complex [178]. This study provides a significant connection between nuclear architecture components and cancer progression. The morphology of the nucleus is also dependent on alteration in nucleoli architecture during tumorigenesis. Nucleolar component-targeted therapeutic drugs, namely Doxorubicin, Mitomycin [179], Cisplatin, Etoposide [180], Actinomycin D [181], are emerging for the treatment of various cancers, including breast, bladder, thyroid, hematological cancers, sarcomas, head and neck cancers. Doxorubicin, Etoposide and Mitomycin are RNA polymerase transcription targeting drugs that inhibit the tumor cells via selective inhibition at different interfaces in the transcription complex. Both Doxorubicin and Etoposide bind to topoisomerase II to arrest tumor growth. Actinomycin D is a DNA-binding drug that intercalates into GC rich DNA regions and inhibits the polymerase I transcription. Similarly, Cisplatin is a DNA-intercalating agent which forms an irreversible interstrand crosslink to guanine and adenine residues of the DNA strand. A new class of targeting rDNA, DNA aptamers and naphthalene diimides, have shown significant potency in inhibiting breast and lung carcinoma proliferation [182,183].

Such highly effective drugs restore the nuclear structure and could be also used to reveal the structural and functional connection of the nucleus. Advances in understanding the mechanism of nuclear structure-based pathophysiology will serve as powerful tool for increasing survival rate and reducing the treatment costs for many fatal diseases.

## 5. Conclusions and Future Perspectives

The molecular mechanisms orchestrating nuclear morphology and their connection to disease development still need to be elucidated clearly. In this review, we have summarized various factors that are contributing to maintaining nuclear morphology and architecture in eukaryotic cells. In fact, the factors described above have profound effects on the structure and function of chromatin, showing correlations with the resulting gene expression and chromosome stability. Moreover, these factors act as a bridge between the cytoskeleton and nucleoskeleton, thus emerging as a promising signal transduction between the nucleus and cytoplasm. It was established that the abnormalities in nuclear morphology could be due to mutations, abnormal gene expression, altered signal transduction pathways and chromatin architecture as well as aneuploidy. In recent years, questions regarding the molecular mechanisms that regulate nuclear size and shape differently in normal and disease states remain largely unanswered. However, there is clear evidence that highlights the influence of abnormal nuclear morphology on different cellular functions, cell cycle, genomic stability, apoptosis and signal transduction pathways. The current literature supports the use of nuclear morphological abnormalities for the early diagnosis of diseases and is beginning to shed light on the use of theranostic approaches for the treatment of diseases. The identification of these nuclear morphological abnormalities-related targets for therapeutic intervention could be promising for personalized cancer treatment and eradication of life-threatening diseases.

## Data Availability

Not applicable.
